# Sex/gender reporting and analysis in Campbell and Cochrane systematic reviews: a cross-sectional methods study

**DOI:** 10.1186/s13643-018-0778-6

**Published:** 2018-08-02

**Authors:** Jennifer Petkovic, Jessica Trawin, Omar Dewidar, Manosila Yoganathan, Peter Tugwell, Vivian Welch

**Affiliations:** 1 Bruyère Research Institute, 85 Primrose Ave, Ottawa, Ontario K1N 6M1 Canada; 2 Ottawa Hospital Research Institute, Centre for Practice-Changing Research, Mailbox 201B, The Ottawa Hospital - General Campus, 501 Smyth Road, Ottawa, Ontario K1H 8L6 Canada; 3 Ottawa Hospital Research Institute, Clinical Epidemiology Program, Ottawa, K1Y 4E9 Canada; 4 University of Ottawa, School of Epidemiology and Public Health, Ottawa, K1H 8M5 Canada

**Keywords:** Systematic reviews, Sex and gender, Health equity

## Abstract

**Background:**

The importance of sex and gender considerations in research is being increasingly recognized. Evidence indicates that sex and gender can influence intervention effectiveness. We assessed the extent to which sex/gender is reported and analyzed in Campbell and Cochrane systematic reviews.

**Methods:**

We screened all the systematic reviews in the Campbell Library (*n* = 137) and a sample of systematic reviews from 2016 to 2017 in the Cochrane Library (*n* = 674). We documented the frequency of sex/gender terms used in each section of the reviews.

**Results:**

We excluded 5 Cochrane reviews because they were withdrawn or published and updated within the same time period as well as 4 Campbell reviews and 114 Cochrane reviews which only included studies focused on a single sex. Our analysis includes 133 Campbell reviews and 555 Cochrane reviews. We assessed reporting of sex/gender considerations for each section of the systematic review (Abstract, Background, Methods, Results, Discussion). In the methods section, 83% of Cochrane reviews (95% CI 80–86%) and 51% of Campbell reviews (95% CI 42–59%) reported on sex/gender. In the results section, less than 30% of reviews reported on sex/gender. Of these, 37% (95% CI 29–45%) of Campbell and 75% (95% CI 68–82%) of Cochrane reviews provided a descriptive report of sex/gender and 63% (95% CI 55–71%) of Campbell reviews and 25% (95% CI 18–32%) of Cochrane reviews reported analytic approaches for exploring sex/gender, such as subgroup analyses, exploring heterogeneity, or presenting disaggregated data by sex/gender.

**Conclusion:**

Our study indicates that sex/gender reporting in Campbell and Cochrane reviews is inadequate.

## Background

Integration of sex/gender in systematic reviews is important for understanding the applicability of evidence. An increasing number of health researchers are accounting for sex/gender in their studies in recognition of this importance [1, 2]. This shift is due, in part, to an increasing number of research funders and scientific journals requiring researchers to account for sex and gender in their research [3].

Sex refers to the biological attributes, such as physiological characteristics, that generally distinguish males and females. Gender refers to the socially constructed roles, behaviors, and identities of girls, women, boys, men, and gender-diverse individuals. Gender influences the way people act and interact with each other, how they view themselves and others, and the distribution of power within a society. Sex and gender can interact with other determinants of health to influence health status and the effectiveness of interventions.

The risk due to underrepresentation of population subgroups, including women, is well known in primary research. For example, the safety of interventions may differ depending on sex/gender; between 1997 and 2000, eight out of 10 drugs withdrawn by the Food and Drug Administration (FDA) in the USA were the result of greater risks of adverse events for women [4]. In 2013, new information about the adverse effects for a drug to treat insomnia, particularly for women, required the FDA to recommend lower starting doses because of the increased risk of next-morning impairment [5]. Additionally, the benefit of an intervention may vary depending on sex/gender. A systematic review of carotid endarterectomy found that the benefits of surgery were greater for men than women and when stenosis was 50–69% there was no evidence of benefit for women [6]. This information is important as it could lead to separate recommendations, preventing women from undergoing surgery for little to no benefit.

The proportion of female participants in randomized controlled trials (RCTs) has been increasing, in part due to increasing FDA requirements [7]. However, this has not led to an increase in analyses of outcomes by sex/gender. Recently, a study assessing gender in a sample of RCTs published in leading medical journals indexed in PubMed found that while sex/gender data were available to the authors of the studies, they were not often analyzed or discussed. The authors reported that none of the included studies described a priori methods to assess sex/gender differences, only 8 of the 57 included studies presented sex-disaggregated data, and none of these discussed these results or provide reasons for differences in outcomes [8]. Similarly, a review of 100 Canadian RCTs found that only 6% of studies conducted subgroup analyses across sex/gender of which only one discussed the challenges with such analyses and the implications of the findings for clinical practice [9].

Cochrane is an international network of researchers, patients, and health professionals who aim to produce and maintain systematic reviews of healthcare interventions. A related organization, the Campbell Collaboration, produces systematic reviews of interventions in crime and justice, disability, education, international development, and social welfare. The Campbell and Cochrane Equity Methods Group was established in 2007 to encourage authors of systematic reviews to consider whether the effectiveness of the intervention will differ by population subgroup. The Equity Methods Group uses the acronym PROGRESS (Place of residence, Race/ethnicity/culture/language, Occupation, Gender/sex, Religion, Education, Socioeconomic status, Social capital) to help remind systematic review authors to consider whether the intervention may have differential effects across these characteristics [10]. Sex/gender is a key focus of the Campbell and Cochrane Equity Methods Group; (http://methods.cochrane.org/equity/sex-and-gender-analysis); they have developed guidance for systematic review authors, such as briefing notes [11] and a planning tool [12].

The use of systematic reviews in policy and practice decision making is increasing [13, 14]. However, policymakers often cite the lack of context and equity considerations (e.g., including sex/gender information) as barriers to using systematic reviews in decision making [12, 14–16]. A major reason is that despite an increasing desire to incorporate sex/gender considerations in research, sex/gender differences remain underreported [17]. Systematic reviews are limited in their ability to report on sex/gender if the studies included in the review have not reported sex/gender considerations. Thus, it is unsurprising that an assessment of the Canadian clinical practice guidelines found inconsistencies in the degree to which sex/gender considerations are incorporated, with only 35% of guidelines providing specific screening, diagnosis, or management considerations based on sex/gender [18].

Within systematic reviews, considering sex/gender implies reporting not only the population characteristics of the included studies but also providing some insight into the possible sex/gender differences in the prevalence of the condition, the benefit of the intervention, or safety concerns. Often, systematic reviews that have considered sex/gender have done so using a subgroup analysis, but considerations of the applicability of the evidence are also important. For example, if the authors of the systematic review note that the included studies are gender imbalanced, then the results of the review may not be universally applicable and this should be reported. If the authors fail to mention this, it presents a missed opportunity to address potentially important issues related to sex/gender and our knowledge of benefits/harms for all those who may receive the intervention.

This project began as a partnership with the CIHR Institute of Gender and Health (IGH) with the aim of developing a Cochrane Corner to highlight Cochrane systematic reviews which have considered or assessed sex/gender. The goal of this Cochrane Corner was to increase awareness of the Cochrane Library among those involved in sex/gender and health research while also increasing awareness of sex/gender-based analyses among the Cochrane community. The Cochrane Corner is now available on the Campbell and Cochrane Equity Methods Group’s website: https://methods.cochrane.org/equity/igh-cochrane-corner.

We reviewed systematic reviews published in the Campbell and Cochrane Libraries to assess the extent to which sex/gender is reported and analyzed. For the purposes of this study, we use the term sex/gender to refer to all concepts of sex and/or gender.

## Methods

We screened the full text of every systematic review published in the Campbell Library (*n* = 137 as of September 2017). For the Cochrane Library, we used the advanced search option within the Archie database to select only reviews which used one of our sex/gender search terms (“sex,” “gender,” “male,” “women,” “boys,” “girls”) in at least one of the following review sections: title/abstract, introduction, methods, results, or discussion (*n* = 674) (total number of reviews published between August 1, 2016, and July 31, 2017, was 1373). We included only the previous year of Cochrane reviews since this resulted in a large sample of reviews representing all of the Cochrane Review Groups. However, since the Campbell Library has a smaller number of published reviews, we decided to include all reviews.

We developed and pre-tested a data extraction form in Excel using the European Association of Science Editors (EASE) Sex and Gender Equity in Research (SAGER) guidelines and our previous work assessing reporting of sex/gender in a sample of randomized controlled trials [19, 9]. We assessed the full text of each review for reporting of sex/gender. One author (one of JT, OD, and MY) independently extracted the text for each section in which sex/gender was reported for each systematic review. A second author (JP) randomly selected 10%, using a random number generator, for independent data quality verification. We classified each mention of sex/gender in each section of the review as descriptive assessment of reporting and analysis (e.g., mention of sex/gender), analytic approaches (e.g., subgroup analyses), and judging applicability (e.g., explicit statement about the applicability of the results).

For those studies which did not report on sex/gender in the abstract, we assessed whether they reported on sex/gender in other review sections. Similarly, we assessed whether reviews that mentioned sex/gender in the methods section also reported sex/gender in the results or discussion. We have summarized the data using descriptive statistics and 95% confidence intervals.

We categorized reviews which stated that recruitment was limited to one sex as “single sex” and excluded these from our analysis. We made this distinction based on how the systematic review authors described eligible studies, particularly in the “types of participants” section of the review. When eligibility was open to more than one sex/gender, we considered the review to be “mixed sex.” For example, we classified a review of interventions for female breast cancer survivors as “single sex” and excluded it. However, in some reviews, although a condition may be sex-specific, the authors included interventions in which multiple sex/genders were eligible. For example, a review of interventions to reduce female genital mutilation included studies in which any members of communities practicing female genital mutilation or cutting were recruited (e.g., men and women and community leaders). In these cases, we classified the reviews as “mixed sex” and included them in our analysis. Finally, to compare sex/gender reporting in Campbell and Cochrane reviews, we assessed the reviews published in 2016–2017 since reporting guidelines have changed considerably over the years and we felt it was inappropriate to compare Campbell reviews from 2004 with Cochrane reviews from 2016 [20–24]. Since the number of Campbell reviews published in 2016–2017 is small, we compared this sample to Cochrane reviews published by review groups in which the interventions assessed may be more comparable to the reviews published by Campbell. These groups included the Consumers and Communication (CCG), Effective Practice and Organization of Care (EPOC), and Cochrane Public Health (CPHG) review groups. The Campbell Collaboration publishes reviews of interventions addressing broad social issues, such as education, crime and justice, and international development. As such, the questions are often broader and may require the inclusion of a range of study designs. The Cochrane CCG publish reviews of interventions to assess the way people interact with healthcare professionals, services, and researchers; EPOC publish reviews of interventions assessing professional practice and the delivery of health services; and CPHG publish systematic reviews of interventions at the population level to assess health, wellbeing, learning, and social outcomes. For these reasons, we decided that Campbell reviews were most comparable to these groups than other Cochrane review groups which assess interventions to prevent, treat, or manage specific health conditions.

## Results

We screened 137 Campbell reviews published between May 2004 and July 2017 and 674 new and updated Cochrane reviews published between August 2016 and July 2017. Five Cochrane reviews were excluded because they were withdrawn or published and updated (or published with a correction) within the same time frame.

We classified 114 Cochrane reviews and 4 Campbell reviews as single sex because they restricted eligibility to studies that included women only (*n* = 114) or men only (*n* = 4). Of the 114 Cochrane reviews, 27 (male *n* = 1; female *n* = 26) were considered sex-specific (e.g., interventions for vasectomy), 3 restricted eligibility to a single sex but mentioned the opposite sex/gender (e.g., interventions for assisted reproductive technologies that recruited only females but mentioned male factor infertility), and 9 restricted eligibility to a single sex with no mention of the opposite sex/gender even though the condition is not sex-specific (e.g., breast cancer reviews that only included women). There were 75 reviews that we considered to be maternal health focused (e.g., breastfeeding, pregnancy) where only 4% of applicable reviews mentioned the sex of the fetus or infant and 60% of applicable reviews mentioned partner, family, couple, or father involvement. The four Campbell single-sex reviews focused on interventions for female victims of intimate partner abuse, interventions for men who abuse their female partner, interventions for women’s empowerment, and treatment for male sexual offenders.

**Fig. 1:**
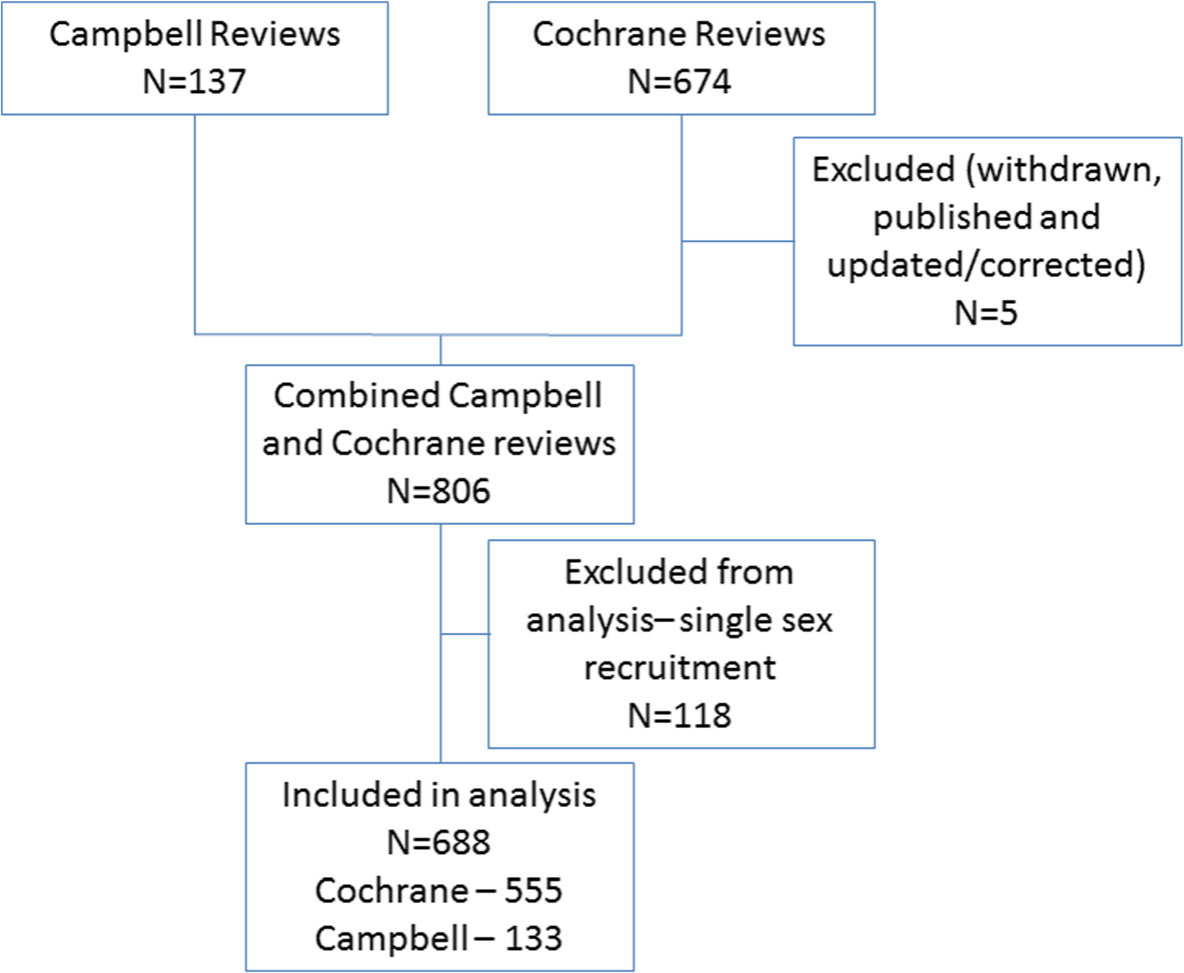
Study flow diagram

Therefore, the total number of Cochrane reviews included in our analysis is 555 and we have included 133 Campbell reviews. The study flow diagram is described in Fig. 1. The characteristics of the systematic reviews included in this study are presented in Table 1, and the sex/gender reporting by review section is presented in Table 2.

**Table 1:** Characteristics of the included systematic reviews

	Campbell	*N* (%)	Cochrane	*N* (%)
Review groups	Crime and justice	41 (29.9)	Acute Respiratory Infections Group	13 (1.9)
	Education	28 (20.4)	Airways Group	27 (4.0)
	International development	25 (18.2)	Anesthesia, Critical and Emergency Care Group	18 (2.7)
	Social welfare	59 (43.1)	Back and Neck Group	3 (0.5)
	Nutrition	1 (0.7)	Bone, Joint and Muscle Trauma Group	6 (0.9)
			Breast Cancer Group	10 (1.5)
			Childhood Cancer Group	8 (1.2)
			Colorectal Cancer Group	5 (0.7)
			Common Mental Disorders Group	10 (1.5)
			Consumers and Communication Group	3 (0.5)
			Cystic Fibrosis and Genetic Disorders Group	34 (5.0)
			Dementia and Cognitive Improvement Group	10 (1.5)
			Developmental, Psychosocial and Learning Problems Group	11 (1.6)
			Drugs and Alcohol Group	4 (0.6)
			Effective Practice and Organization of Care Group	9 (1.3)
			ENT Group	5 (0.7)
			Epilepsy Group	19 (2.8)
			Eyes and Vision Group	26 (3.8)
			Fertility Regulation Group	7 (1.0)
			Gynecological, Neuro-oncology, and Orphan Cancer Group	12 (1.7)
			Gynecology and Fertility group	33 (4.8)
			Hematological Malignancies Group	7 (1.0)
			Heart Group	27 (3.9)
			Hepato-Biliary Group	18 (2.6)
			Hypertension Group	8 (1.2)
			IBD Group	8 (1.2)
			Incontinence Group	3 (0.5)
			Infectious Diseases Group	9 (1.3)
			Injuries Group	5 (0.7)
			Kidney and Transplant Group	12 (1.7)
			Lung Cancer Group	4 (0.6)
			Metabolic and Endocrine Disorders Group	6 (0.9)
			Methodology Review Group	1 (0.1)
			Movement Disorders Group	3 (0.5)
			Multiple Sclerosis and Rare Diseases of the CNS Group	2 (0.3)
			Musculoskeletal Group	6 (0.9)
			Neonatal Group	23 (3.4)
			Neuromuscular Group	9 (1.3)
			Oral Health Group	20 (2.9)
			Pain, Palliative and Supportive Care Group	59 (8.6)
			Pregnancy and Childbirth Group	51 (7.4)
			Public Health Group	4 (0.6)
			Schizophrenia Group	20 (2.9)
			Skin Group	7 (1.0)
			STI Group	4 (0.6)
			Stroke Group	18 (2.6)
			Tobacco Addiction Group	8 (1.2)
			Upper GI and Pancreatic Diseases Group	8 (1.2)
			Urology Group	4 (0.6)
			Vascular Group	23 (3.6)
			Work Group	5 (0.7)
			Wounds Group	14 (2.0)
Publication year	2004	1 (0.7)		
	2005	5 (3.6)		
	2006	11 (8.0)		
	2007	5 (3.6)		
	2008	18 (13.1)		
	2009	4 (2.9)		
	2010	3 (2.2)		
	2011	9 (6.6)		
	2012	19 (13.9)		
	2013	13 (9.5)		
	2014	11 (8.0)		
	2015	20 (14.6)		
	2016	10 (7.3)	2016	279 (41.4)
	2017	8 (5.8)	2017	395 (58.6)
Single-sex review	Females abused by partners	1 (0.7)	Female breast cancer	10 (1.5)
	Males who abuse partners	1 (0.7)	Gynecology and fertility	45(6.7)
	Male sexual offenders	1 (0.7)	Hemophilia	1(0.1)
	Women’s empowerment	1 (0.7)	Pregnancy, child birth, and breastfeeding	57 (8.5)
			Sterilization	1 (0.2)

**Table 2:** Reporting of sex/gender by review section

	Abstract* *N* % (95% CI)	Background* *N* % (95% CI)	Methods* *N* % (95% CI)	Results* *N* % (95% CI)	Discussion* *N* % (95% CI)
Campbell reviews (*N* = 133)	18 13.5% (8–19)	26 19.5% (13–26)	68 50.8% (42–59)	30 22.6% (15–30)	18 13.5% (8–19)
Cochrane reviews (*N* = 555)	55 9.9% (7–12)	153 27.6% (24–31)	461 83.1% (80–86)	161 29.0% (25–33)	81 14.6% (12–18)
Total	73 10.6% (8–13)	179 26.0% (23–29)	529 76.8% (74–80)	191 27.7% (24–31)	99 14.4% (12–17)

**Fig. 2:**
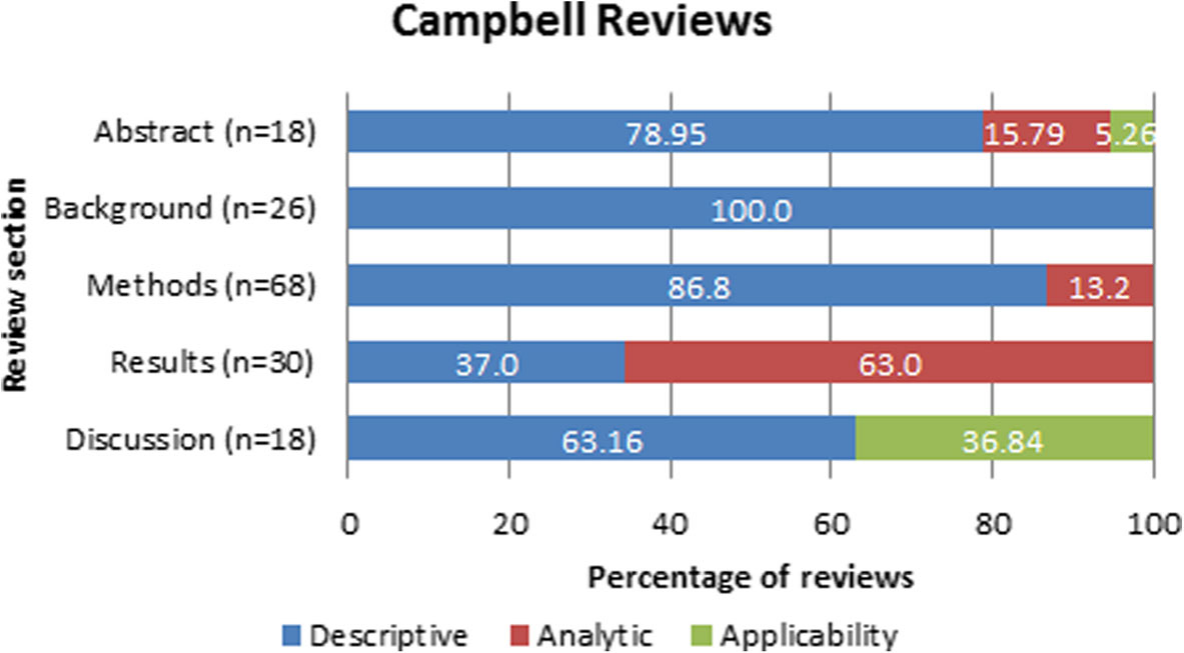
Categories of sex/gender reporting in Campbell systematic reviews

**Fig. 3:**
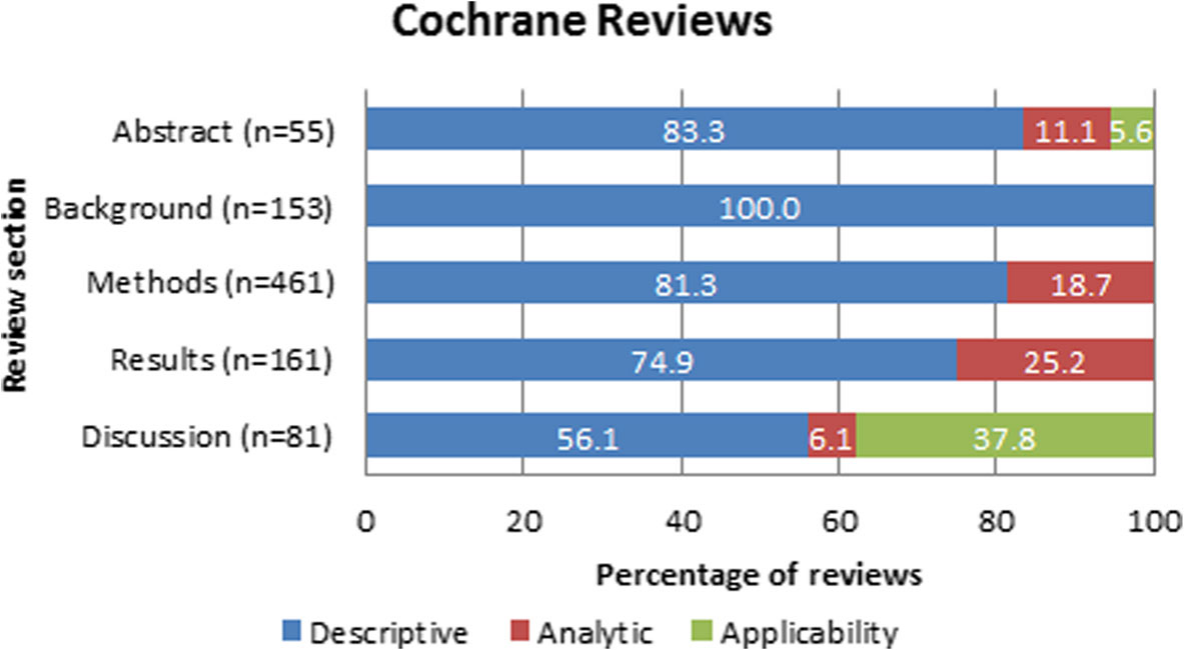
Categories of sex/gender reporting in Cochrane systematic reviews

*When sex/gender terms were used only to describe characteristics (e.g., sex/gender listed as a data extraction item, number of males/females/men/women included in the studies), we did not count these in the above but have included them in Figs. 2 and 3 as “descriptive” mentions of sex/gender

Only 14% (95% confidence interval (CI) 8–19%) of Campbell reviews and 10% (95% CI 7–12%) of Cochrane reviews mentioned sex/gender in the abstract of the review. Of these, 16% (95% CI 10–22%) of Campbell reviews and 11% (95% CI 6–16%) of Cochrane reviews included analytic descriptions of sex/gender in the abstracts. Only one Campbell review reported on the applicability of the evidence with regard to sex/gender (5%, 95% CI 1–9%). The sex/gender reporting of Campbell reviews is presented in Fig. 2 and Cochrane reviews in Fig. 3.

In the background section, 20% (95% CI 13–26%) of Campbell reviews and 28% (95% CI 24–31%) of Cochrane reviews mentioned sex/gender. These were all descriptive, such as reporting the prevalence of the condition or risk factors by sex/gender.

In the methods section, 51% (95% CI 42–59%) of Campbell and 83% (95% CI 80–86%) of Cochrane reviews reported sex/gender considerations. Of these, 87% (95% CI 81–93%) and 81% (95% CI 75–88%) of Campbell and Cochrane reviews respectively mentioned sex/gender in a descriptive way in the methods section. Most often, this was with respect to including sex/gender as data collection items or describing the populations of the included studies. Analytic approaches to sex/gender were reported in the methods section of 13% (95% CI 7–19%) of Campbell and 19% (95% CI 12–25%) of Cochrane reviews, such as planned subgroup analyses as well as investigating population characteristics, such as exploring sex/gender as a source of heterogeneity.

When reported in the methods section, 9.8% (95% CI 5–15%) of Campbell reviews and 20.5% (95% CI 17–24%) of Cochrane reviews also reported sex/gender in the results section and 6.8% (95% CI 2–11%) of Campbell reviews and 12.1% (95% CI 9–15%) of Cochrane reviews reported sex/gender in the discussion section.

In the results sections, 23% (95% CI 15–30%) of Campbell and 29% (95% CI 25–33%) of Cochrane reviews reported on sex/gender. Of these, 34% (95% CI 26–43%) of Campbell and 75% (95% CI 68–82%) of Cochrane reviews provided a descriptive report of sex/gender, such as the characteristics of the included populations, and 66% (95% CI 57–74%) of Campbell reviews and 25% (95% CI 18–32%) of Cochrane reviews reported analytic approaches for sex/gender. The analytic approaches included subgroup analyses (tests for interaction across sex/gender), assessment of heterogeneity (restricting to one sex/gender and assessing whether the results are consistent with the overall effects), or presentation of sex-disaggregated data (without testing for interactions). When reported in the results section of the review, 6.8% (95% CI 2–11%) of Campbell reviews and 9.2% (95% CI 7–12%) of Cochrane reviews also reported sex/gender in the discussion section.

For the discussion section, 14% (95% CI 8–19%) of Campbell and 15% (95% CI 12–18%) of Cochrane reviews reported on sex/gender. Of these, 37% (95% CI 29–45%) of Campbell reviews and 38% (95% CI 30–46%) of Cochrane reviews provided judgments about the applicability of the evidence for sex/gender in the discussion section of the reviews. Examples of the considerations of sex/gender as reported in each section of the review are provided in Table 3.

**Table 3:** Examples of reporting in each systematic review section

Review section	Examples
Abstract	“Most participants in the studies included in this review were male. None of the studies reported outcomes on the basis of sex, preventing any exploration of differences related to this variable. Consideration of sex as a factor influencing response to withdrawal treatment would be relevant research for selecting the most appropriate type of intervention for each individual” [33] “To assess the effectiveness in women and the safety in men of concurrent antibiotic treatment for the sexual partners of women treated for bacterial vaginosis” [34] “In addition, the gender of the facilitator seems to play an important role, since women prefer to discuss private issues with somebody of the same sex” [35].
Background	“The prevalence of AAA increases with age and occurs much more frequently in men than women” [36]. “In general, males are more likely to be dropouts than females (9.8% vs. 7.7%), but teenage pregnancy and parenthood are particularly strong risk factors for young women, especially in the United States” [37] “In the USA alone, gallstones are present in 8% to 20% of the population by the age of 40 years, and are more likely to develop in women than in men by a ratio of between 2 and 3 to 1” [38]
Methods	“We considered performing subgroup analyses to establish effectiveness relative to gender, chronicity, age or stroke severity (respectively men versus women; early (less than one year post-stroke) versus late (more than one year post-stroke); young adults versus older; mild/moderate versus severe stroke, if sufficient data were available” [39]. “We would have considered type of intervention and duration of intervention as well as gender of psychiatrist and patient, education in the UK versus non-UK trained psychiatrists” [40]. “Although it was planned to disaggregate studies by gender where possible, we found a gaping lacuna of gender-relevant evidence and were unable to quantitatively examine differential impacts for women and men, as is discussed in our section on opportunities for further research” [41].
Results	“There was no indication of a differential effect in serious adverse events, withdrawals due to adverse effects or changes in blood pressure at one year. However, there were too few women to make any conclusions” [42]. “When we pooled Bryson 1983 and Kinghorn 1986b, and considered men and women separately, for males there is a difference in the duration of symptoms after treatment with acyclovir (MD −2.10 days, 95% CI − 4.28 to 0.09; 2 RCTs, 33 men, I2 statistic = 0%). In females there was high heterogeneity between the two trials included in the meta-analysis and it did not show any statistical difference between those taking acyclovir and those taking placebo (MD −4.13 days, 95% CI − 10.15 to 1.89; 2 RCTs, 49 women, I2 statistic = 71%). However overall, we did not observe any statistical difference between men and women (Test for subgroup differences: Chi2 = 0.39, *P* = 0.53) for the duration of symptoms from onset of treatment” [43]. “Whether officially members of certified POs or not, women involved in certified production are often reported to be disadvantaged in terms of both the benefits they receive and in their influence over decision making within the certified [Producer Organization] POs. For instance, certification-related training may in theory be open to all PO members, in practice, however, women are reported to be less likely to participate, possibly because training is not tailored to their needs and agenda” [44].
Discussion	“One study examined pregnant women (Powell 2011); as it is unknown how FeNO levels are affected during pregnancy, extrapolation of this review to pregnancy is limited. Furthermore, less than 50% of women in this study were on ICS at baseline. As the participants in the rest of the studies were on ICS, results of this review should not be extrapolated to adults with asthma who do not require daily ICS to control their symptoms” [45] “…the absence of follow-up studies assessing the long-term impact of a bulging fontanelle after supplementation; and the finding of a potentially harmful effect among female infants, additional research is warranted before a decision can be reached regarding any policy recommendations for this intervention” [26]. “Although many of the included studies provided some information about gender differences in impact, relatively few explored how the impact of TVET interventions on young women and men might then vary according to other populations characteristics, such as age, socio-economic status, and location” [46].

We assessed whether reporting of sex/gender considerations in the abstract of the systematic review is an indicator of its reporting in other review sections. For Campbell reviews, only 12% reported on sex/gender in the abstract. However, 62% (95% CI 54–71%) reported sex/gender considerations in other sections of the review without mentioning them in the abstract. In the results section, 47% of Campbell reviews (95% CI 39–56%) reported sex/gender without reporting it in the abstract.

For Cochrane reviews, while 10% reported sex/gender in the abstract, 86% (95% CI 83–89%) reported sex/gender in another section of the review without reporting it in the abstract. This includes 23% (95% CI 19–26%) that reported sex/gender in the results section but not in the abstract and 11% (95% CI 6–17%) of Campbell and 11% (95% CI 9–14%) of Cochrane reviews that reported sex/gender in the discussion but not the abstract.

Of the 51% (95% CI 42–59) of Campbell reviews reporting sex/gender in the methods, 81% (95% CI 75–88%) were descriptive and 19% (95% CI 12–25%) were analytic. However, in the results section, 37% (95% CI 29–45%) of the instances of reporting sex/gender were descriptive while 63% (95% CI 55–71%) were analytic. For the Cochrane reviews in our sample, 83% (95% CI 80–86%) reported sex/gender in the methods of which 81% (95% CI 75–88%) were descriptive and 19% (95% CI 12–25%) were analytic. In the results section, 75% (95% CI 18–32%) were descriptive and 25% (95% CI 18–32%) were analytic.

**Fig. 4:**
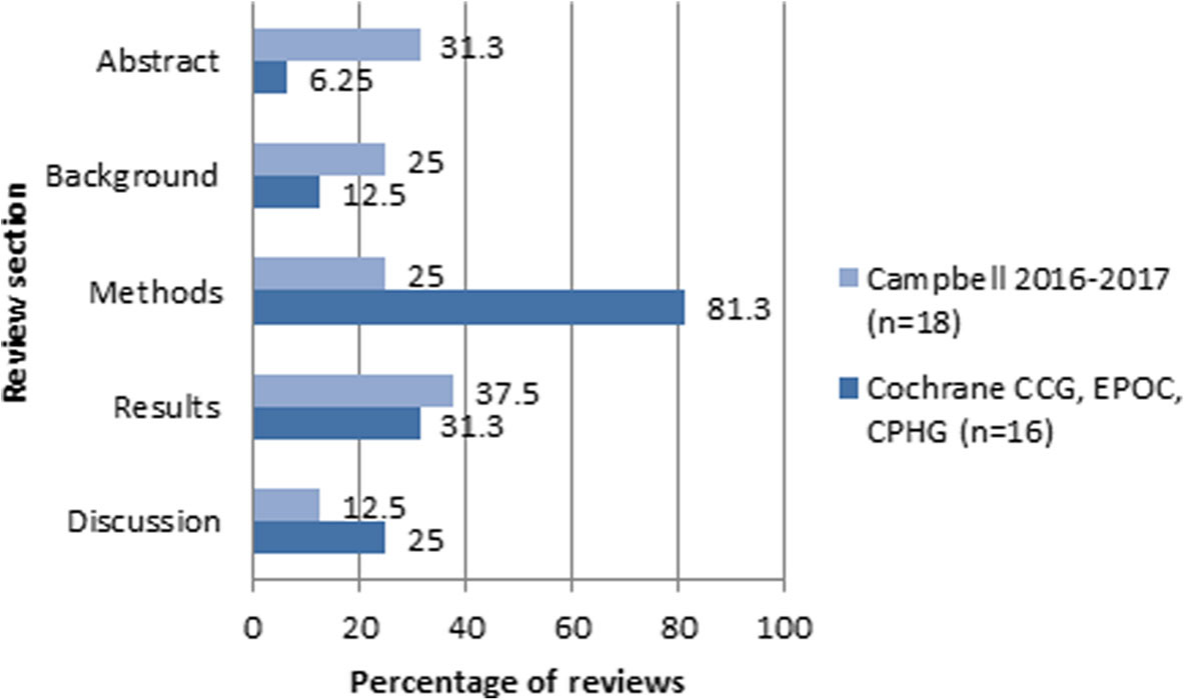
Reporting of sex/gender by review section, 2016–2017 Campbell and Cochrane CCG, EPOC, and CPGH reviews

Finally, we compared the sex/gender reporting of the Campbell reviews published in 2016–2017 (*n* = 18) to the reviews published by the Cochrane CCG, EPOC, and CPHG (*n* = 16) (Fig. 4). Campbell reviews in this time period included more sex/gender considerations in the abstract (31.1% vs. 6.3%), background (25% vs. 12.5%), and results (37.5% vs 31.3%) compared to Cochrane while the Cochrane reviews reported more sex/gender considerations in the methods (81.3% vs 25%) and discussion (25% vs 12.5%) sections.

## Discussion

Overall, the methods sections included the most reporting of sex/gender in both Campbell (50.8%) and Cochrane (83.1%) reviews. The majority of these were descriptive considerations of sex/gender, such as the collection of sex/gender data. A previous study of systematic reviews indexed in MEDLINE in 1 month in 2004 found that almost half of systematic reviews described the sex/gender of the included populations but only 4% assessed sex/gender differences or included sex/gender when making judgements about the applicability or the implications of the evidence [25]. A study of sex/gender reporting in RCTs in 2013–2014 found that 98% of trials report the sex/gender characteristics of their included population which indicates that there is an opportunity for reporting within reviews to improve [9].

We found that 62% of Campbell reviews and 86% of Cochrane reviews did not report sex/gender in the abstract but included sex/gender considerations in a later section. This included 47% of Campbell reviews and 23% of Cochrane reviews which reported sex/gender considerations in the results section. This is an important finding since those who use systematic reviews in decision making often rely on the abstract to make a decision about whether to access the full text. Important sex/gender-related evidence, including findings of no differences in effectiveness based on sex/gender, may be missed.

This sample of systematic reviews presents some important evidence regarding sex/gender differences. For example, one review found statistically significantly higher risk of neonatal mortality among female infants for vitamin A supplementation at 12 months follow-up [26]. Another review reported that the risk of stroke or death following carotid endarterectomy for symptomatic carotid stenosis was higher among women and the number needed to treat to prevent one stroke is four times higher for women than men [6].

There has been debate about the impact of the lack of sex/gender information in systematic reviews. When the studies included in a systematic review have not considered sex/gender differences, it represents an applicability issue, but this could also be considered a risk of bias. The Cochrane Risk of Bias tool was developed to help systematic review authors consider the potential limitations of the studies included in their review [27]. It refers to systemic error because replication of the study would produce the same incorrect result. If trialists and the systematic review authors fail to consider potential differences in the effectiveness of an intervention for men and women, this may present a risk of bias, since users of the review are unable to accurately assess for whom the intervention is effective, or not [28]. At the very least, reporting bias could occur if only some included studies provide sex-disaggregated data and therefore any sex-based analysis is only based on that subset of data [11].

Two reviews included in our study did mention sex/gender as a potential source of bias. The first review assessed mobile health clinics but was focused on women and children’s health outcomes and therefore excluded studies in which services were offered exclusively to men [29]. The authors stated that this may have biased the estimate of the effectiveness of mobile health clinics in improving access to health care within underserved communities. The second reported that one included study which assessed postoperative pain was potentially biased because of an uneven distribution of male and female participants between groups. This was a small study with three arms assessing different timing of ibuprofen provided pre- or postoperatively for orthodontic pain. One study arm had more than twice as many female participants while the third had more than three times as many males. Since previous studies have shown evidence of a possible gender-based difference in pain, the review authors reported this as a potential source of bias [30, 31].

The impact of a lack of sex/gender consideration and potential bias resulting from a lack of consideration of sex/gender will depend on the review question and the characteristics of the studies included. For example, for some health conditions, a systematic review including studies with a majority of women may be acceptable, although the rationale should be described as well as any applicability implications. There are certain questions in which sex/gender could be particularly important, such as those about safety (e.g., pharmacokinetics, surgery) or implementation and programmatic effectiveness. Additionally, the analysis of sex/gender differences carries with it the risk of spurious findings if it is one of multiple analyses. We suggest that authors consider and justify all planned subgroup analyses on the basis of prior literature and advice from content experts and other stakeholders. The absence of evidence of a difference in effect is not justification to assume there is no difference. We propose that systematic review authors should consider population characteristics, such as sex/gender, as well as other potentially important factors, using the PROGRESS-Plus acronym and plan to present disaggregated data, when possible, or justify why these considerations are unnecessary.

Currently, Cochrane and the Campbell Collaboration have no specific policy on the reporting of sex/gender in systematic reviews. However, Cochrane has endorsed the SAGER guidelines developed by the European Association of Science Editors [32]. These guidelines aim to provide guidance on reporting of sex and gender information in study design, data analyses, results, and interpretation of the findings [19]. As of February 2017, 92 individuals/organizations have endorsed the SAGER guidelines.

A pilot study assessing Cochrane authors’ opinions of “briefing notes” for including sex/gender in systematic reviews found that authors were receptive to guidance on improving this aspect of their systematic review. However, they noted that depending on the review question, the extent of sex/gender analysis required and the appropriateness and feasibility will vary [11]. Respondents agreed that sex/gender considerations improve the usefulness of systematic review results for end users, such as policy makers and other decision makers.

Our study assessed sex/gender reporting in all Campbell reviews published as of July 2017 and all new and updated Cochrane reviews published between August 2016 and July 2017. This included a wide variety of systematic reviews published with 5 Campbell coordinating groups and 52 Cochrane review groups. This represents at least one review from each active group as of July 2017. However, our study has some limitations. First, we did not screen all Cochrane reviews published during this period and instead searched for key terms reported in each section of the review. This approach may have missed some reviews which only used terminology that implies sex/gender, such as father or mother, without stating it directly. However, we tested this and did not identify any additional reviews published within the time period sampled. Our preliminary work suggested that a sex/gender-specific term is typically used in at least one section of the review, if consideration of sex/gender is included in the review. For gender-diverse terms, we assumed that since the terminology is rapidly expanding, systematic review authors would have used the term “gender” within their review and therefore these reviews would have been included in our sample. We did not conduct data extraction in duplicate and instead performed a random 10% quality check. This could have resulted in missed instances of sex/gender reporting, but since the quality check had almost 100% agreement, we are confident that the risk is low. Additionally, we did not assess whether the terms “sex” or “gender” were used appropriately by the review authors. Instead, we captured any use of any term related to sex or gender as used by the systematic review author. Therefore, we are unable to report whether these terms are used correctly or consistently in systematic reviews.

We identified 117 systematic reviews that restricted eligibility to studies that focused on a single sex. We did not assess whether these reviews limited recruitment appropriately so we cannot comment on whether these reviews have missed the opportunity to assess potential sex/gender-related differences in the effectiveness of the intervention or to report on implications for the applicability of their findings.

Finally, some systematic review authors may consider sex/gender at the review outset but the team may make a decision that these issues are not relevant and therefore do not get report on them in the review. A limitation of our current study is that while these types of considerations may occur, we are limited by the reporting of the systematic reviews.

An ongoing challenge for systematic review authors is the inconsistent and evolving terminology used in primary studies (and elsewhere). The terms sex and gender are often used interchangeably or incorrectly which can make interpretation of data difficult for those using research results. In this study, we did not assess whether the terms were used correctly or consistently within Campbell and Cochrane reviews. However a recent review of sex/gender considerations in Canadian clinical practice guidelines found that only 25% of those that reported sex- or gender-specific considerations for screening, diagnosis, or management used the terms sex and gender appropriately [18]. Additionally, intersectionality, or the interrelationship of sex and gender and other personal characteristics, such as age, race, and socioeconomic status, has emerged as an important consideration [17]. These intersections require some consideration to understand how interventions will work in practice.

## Conclusions

The absence of availability of sex/gender disaggregated data from primary studies and evidence syntheses affects our ability to make relevant policies and programs and affects our ability to reduce health inequities. Since the use of systematic reviews is encouraged for policymaking, an assessment of potential sex/gender-related differences in intervention effectiveness and safety is critically important. Ideally, the increased interest in and emphasis on the importance of sex/gender in research from funders and journal editors for all research, from biomedical research to clinical trials and systematic reviews, will result in improved reporting of these considerations. We hope that if we assess Campbell and Cochrane systematic reviews in 5 years the reporting of sex/gender similarities and differences will be improved.

## Abbreviations

CCG: Consumers and Communication Group (Cochrane)
CI: Confidence interval
CIHR: Canadian Institutes of Health Research
CPHG: Cochrane Public Health Group
EASE: European Association of Science Editors
EPOC: Effective Practice and Organization of Care
FDA: Food and Drug Administration
IGH: Institute of Gender and Health
PROGRESS: Place of residence, Race/ethnicity/culture/language, Occupation, Gender/sex, Religion, Education, Socioeconomic status, Social capital
RCT: Randomized controlled trial
SAGER: Sex and Gender Equity in Research
